# Editorial: Innovations in measurement and evidence for healthy aging

**DOI:** 10.3389/fmed.2023.1347385

**Published:** 2023-12-13

**Authors:** Marcela Agudelo-Botero, Claudio Alberto Dávila-Cervantes, Liliana Giraldo-Rodríguez

**Affiliations:** ^1^Centro de Investigación en Políticas, Población y Salud, Facultad de Medicina, Universidad Nacional Autónoma de México, Mexico City, Mexico; ^2^Facultad Latinoamericana de Ciencias Sociales (FLACSO, Sede México), Mexico City, Mexico; ^3^Instituto Nacional de Geriatría, Mexico City, Mexico

**Keywords:** aging, measurement, health, policy, older people

The World Health Organization (WHO) has declared the decade of Healthy Aging (2021–2030), in alignment with the 2030 Sustainable Development Goals (SDO) to ensure that all older people can fulfill their potential with dignity and equality in appropriate environments. Healthy aging represents a continuous process to develop and maintain the functional capacity that allows wellbeing in old age (1). Functional capacity is composed of a person's intrinsic capacity (combination of all physical and mental capacities), the characteristics of the environment (factors in the outside world that form the context of life), and the interactions between the person and these characteristics (2, 3).

Maintaining functional capacity in older people is a public health priority, and some of the greatest challenges are related to measuring and estimating indicators that reflect how people age, the trajectories of functional and intrinsic capacity, and the impacts of policies and actions that promote healthy aging (1–3).

The objective of this Research Topic was to highlight the various ways of seeing and understanding healthy aging in broad and heterogeneous contexts, in such a way that it serves for the transfer of knowledge, evidence for future research, and for the generation of interventions that favor healthy aging and wellbeing in old age.

This Research Topic brought together a total of 18 articles of the highest quality in which 108 authors participated. The works developed provide valuable results, where enriching sources of information and analysis techniques were used. These publications address current issues surrounding the older adult population in different geographic locations and understanding healthy aging, from objective physical and mental measures to aspects of mental health and wellbeing. Below is a summary of each of these works (according to chronological order of publication).

- Pumpho et al. developed a mobile application called Walking Think that allows easy interaction with users to record the Timed Up and Go test (TUG) duration while performing walking simultaneously with a cognitive dual task. The authors demonstrated that the mobile application is a valid tool to measure the TUG and TUG-subtraction duration. The TUG test was able to distinguish between faller and non-faller older peoples, with high sensitivity and specificity.
- Gui et al. aimed to predict metabolic syndrome using obesity- and lipid-related indices in middle-aged and elderly Chinese adults. The authors used a national cohort study that consisted of 3,640 adults (≥45 years) and found that a total of 13 obesity- and lipid-related indices were independently associated with metabolic syndrome risk.
- E et al. explored the mediational effects of social support between internet use and health among older adults in China, from the 2021 Chinese General Social Survey. The authors recommend that the government should take compelling measures to encourage and promote the use of the internet among older adults and to obtain social support to improve their health status.
- Wu et al., based on the China Health and Retirement Longitudinal Study (CHARLS), performed a cohort analysis (2011/2012–2015) with the objective to illustrate the relationship between the waist-to-height ratio and the incidence of hypertension in middle-aged and older adult women in China.
- Guo et al. developed a system of indicators to measure the risk of social disablement in China, which involves complex multidimensional variables. The authors found that the risk of social disability in China is generally at a moderately high level and that the risk of disability varies considerably both between and within regions and provinces.
- De la Vega Martínez et al. performed a secondary analysis of the National Survey on the Effects of COVID-19 on the Wellbeing of Mexican Households. The authors analyzed the prevalence of food insecurity and its association with depressive and anxiety symptoms in older Mexican adults during the COVID-19 pandemic.
- Kozela et al. assessed the predictive performance of the Healthy Aging Scale for all-cause mortality in middle-aged and older adults. Data from the Polish and Czech Health Alcohol and Psychosocial factors in Eastern Europe prospective cohorts (2002–2005) were used.
- Wu et al. conducted the first study to evaluate the relationship between sarcopenia index (SI) and all-cause mortality in middle-aged and older Chinese adults. Among the cohort, patients with a higher SI had lower mortality, indicating that SI could be an effective marker for assessing mortality in middle-aged and older Chinese adults.
- Segura et al. developed research on the demographic, family, social, personal, and health factors associated with the subjective perception of happiness in older adults in five cities in Colombia. The authors observed that happiness was explained by the absence of risk of depression and little hopelessness, strengthened psychological wellbeing, a perception of high quality of life, and living in a functional family.
- Wang et al. developed a short version of the Chinese Resident Health Literacy Scale focused on older adults in China, and further assessed the reliability and validity of this short version. The data was from a cross-sectional community-based older adults (5,829) health survey conducted in 2020.
- Navarrete-Valladares et al. analized the theoretical-methodological characteristics presented by other studies carried out between 2012 and 2022 on the experience and collective memory of older adults in the face of climate change.
- Lee and Lee investigated the effect of obstructive sleep apnea on hearing ability in a sample that included 3,575 participants in the Korean National Health and Nutrition Examination Survey between 1 January 2019 and 31 December 2020.
- Lee et al. conducted a study in Korea where the authors demonstrated that relative handgrip strength (RGS) is associated with the incidence of CKD in both men and women; therefore, RGS can be used in clinical practice to evaluate renal prognosis.
- Luo et al. designed a study to establish the cut-off value and diagnostic utility of the Ishii test, which gauges the odds of severe sarcopenia from the results of an equation based on age, grip strength, and calf circumference among middle-aged and older adults (≥50 years) from the West China Health and Aging Trend study.
- Yeverino-Castro et al. described the cognitive changes in older adults (>60 years of age or older) with healthy aging from the 2012 and 2015 waves of the Mexican Health and Aging Study.
- Gutiérrez-Barreto et al. evaluated the design of Integrated Care for Older People (ICOPE) through Theory of Change to analyze its possible implementation in Mexico City. The authors propose that ICOPE has the potential to be applied to contexts similar to Mexico, for example, in other lower-middle-income countries.
- Bai and Lu focused on studying the gap in primary health care access between planning evaluation and current utilization for older adults. The authors conducted an empirical study in Dalian city area based on the registration and survey data of community health centers during the COVID-19 pandemic.
- Shen et al. explored the associations of intrinsic capacity (IC), fall risk, and frailty in a Total of 703 hospitalized patients aged 75 years or older that were recruited for this retrospective observational study from Zhejiang Hospital.

In summary, this Research Topic constitutes an important advance in the knowledge of new and innovative measures to study healthy aging, and provides useful information to decision makers at different levels. It also raises the need to collect reliable and quality data while improving data analysis to monitor social and health actions, programs, and policies (4).

## Author contributions

MA-B: Conceptualization, Writing—original draft, Writing—review & editing. CD-C: Writing—original draft, Writing— review & editing. LG-R: Writing—original draft, Writing—review & editing.
